# Biomass and *RRR*-α-tocopherol production in *Stichococcus bacillaris* strain siva2011 in a balloon bioreactor

**DOI:** 10.1186/1475-2859-13-79

**Published:** 2014-06-03

**Authors:** Ganapathy Sivakumar, Kwangkook Jeong, Jackson O Lay

**Affiliations:** 1 Arkansas Biosciences Institute and College of Agriculture and Technology, Arkansas State University, PO Box 639, Jonesboro, AR 72401, USA; 2 College of Engineering, Arkansas State University, Jonesboro, AR 72401, USA; 3 Arkansas Statewide Mass Spectrometry Facility, University of Arkansas, Fayetteville, AR 72701, USA

**Keywords:** Antioxidant, Biomass, Bioreactor, Microalgae, Vitamin E

## Abstract

**Background:**

Green microalgae represent a renewable natural source of vitamin E. Its most bioactive form is the naturally occurring *RRR*-α-tocopherol which is biosynthesized in photosynthetic organisms as a single stereoisomer. It is noteworthy that the natural and synthetic α-tocopherols are different biomolecular entities. This article focuses on *RRR*-α-tocopherol production in *Stichococcus bacillaris* strain siva2011 biomass in a bioreactor culture with methyl jasmonate (MeJa) elicitor. Additionally, a nonlinear mathematical model was used to quantitatively scale-up and predict the biomass production in a 20 L balloon bioreactor with dual variables such as time and volume.

**Results:**

Approximately 0.6 mg/g dry weight (DW) of *RRR*-α-tocopherol was enhanced in *S. bacillaris* strain siva2011 biomass with the MeJa 50 μL/L for 24 hrs elicitations when compared to the control. The R^2^ value from the nonlinear model was enhanced up to 95% when compared to the linear model which significantly improved the accuracy for estimating *S. bacillaris* strain siva2011 biomass production in a balloon bioreactor.

**Conclusions:**

*S. bacillaris* strain siva2011 is a new green microalga which biosynthesizes significant amounts of *RRR*-α-tocopherol. Systematically validated dual variable empirical data should provide key insights to multivariable or fourth order modeling for algal biomass scale-up. This bioprocess engineering should provide valuable information for industrial production of *RRR*-α-tocopherol from green cells.

## Background

*RRR*-α-tocopherol is a lipid soluble small molecule and the biologically active form of natural vitamin E. *RRR*-α-tocopherol is exclusively biosynthesized by photosynthetic organisms or green cells including algae, plants, and cyanobacteria [1-3]. Plant-based products are a primary source of *RRR*-α-tocopherol in the human diet. For example, hazelnut is one of the richest sources of vitamin E [4]. It is known that vitamin E plays an important role in human nutrition as a natural antioxidant. It was recently proposed that vitamin E is active against oxidative stress-related diseases [5]. Reportedly, it suppresses telomerase activity in ovarian cancer cells [6]. Vitamin E enhances IL-2 production, gene expression, and is an effective therapeutic adjuvant [7]. Vitamin E deficiency affects both T and B immune cell functions [8], the α-tocopherol transfer protein (α-TTP) gene, and neurologic dysfunctions. In animal models, vitamin E mixtures inhibit colon, lung, mammary, and prostate carcinogenesis [9], as well as prevent diabetes [10]. Cosmetic industries also extensively use vitamin E in skin care products. In addition, *RRR*-α-tocopherol prolongs the shelf life of meat [11]. Thus, the use of *RRR*-α-tocopherol continues to increase in nutraceuticals [12].

**Figure 1:**
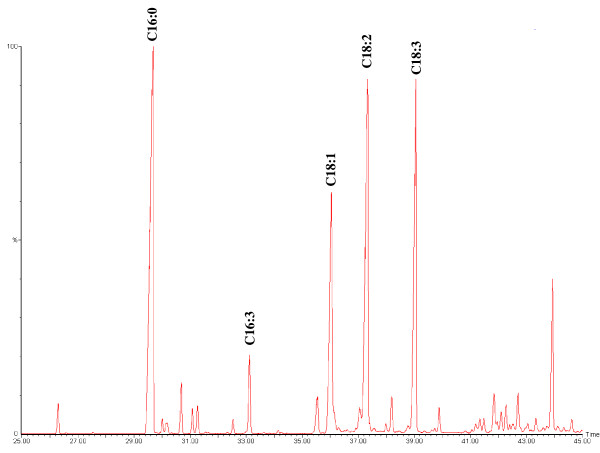
**Gas chromatography mass spectrometry profile of fatty acid methyl esters from *S. bacillaris *strain siva2011, biomass from 20 L bioreactor, working volume 8 L with 0.2% CO**_**2 **_**on day 6.** Methyl palmitate (C16:0), methyl hexadecatrienoate (C16:3), methyl oleate (C18:1), methyl linoleate (C18:2), and methyl linolenate (C18:3).

Bioreactor technology is the key component for the industrial scale production of bioactive small molecules for pharmaceutical applications. For instance, bioreactor technology was successfully developed and scaled-up to 10,000 L for commercial-scale production of ginseng roots used for human health-related applications [13,14]. This reactor configuration has also been tested for *RRR*-α-tocopherol production in lab-scale photosynthetic hazelnut root culture [15]. However, knowledge regarding the bioprocessing of green algal cells for production of *RRR*-α-tocopherol in balloon bioreactors is limited. Previously, *Stichococcus bacillaris* strain siva2011 biomass was scaled-up in a lab-scale balloon bioreactor (4 L - 8 L), and a linear fitting model for predicting scale-up was proposed [16]. The *S. bacillaris* strain siva2011 has unique lipid (Figure 1) [16] and vitamin E metabolisms to lead to bioactive *RRR*-α-tocopherol production. A nonlinear model enables more accurate estimates and should provide insight for quantitatively predicating algal biomass accumulation in a large volume bioreactor. The objectives of this study were to: 1) evaluate *RRR*-α-tocopherol content from *S. bacillaris* strain siva2011 biomass with MeJa elicitation; 2) quantitatively predict *S. bacillaris* strain siva2011 DW in a 20 L bioreactor with simplified stepwise dual variables (time and volume) from 4 L and 8 L data using nonlinear regression. This systematic study could provide insight regarding stepwise nonlinear scale-up of *S. bacillaris* strain siva2011 biomass for *RRR*-α-tocopherol production in a balloon bioreactor.

## Results and discussions

**Figure 2:**
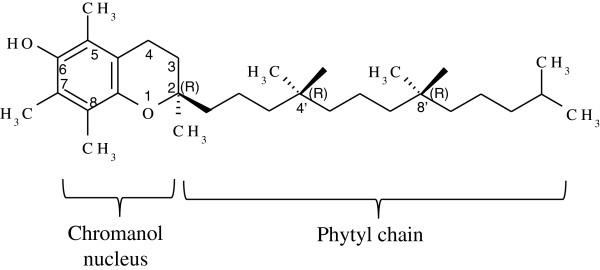
**Chemical structure of *RRR*-α-tocopherol.**

Natural vitamin E occurs in two general forms, namely the tocopherols and tocotrienols, which are collectively called tocochromanols. Each has four distinct isoforms having α, β, γ, or δ substitution on the chromanol. The natural α-tocopherol contains three methyl groups on the chromanol moiety at positions 5, 7, and 8. The phytyl or saturated side chain is attached to the C-2 position of the chromanol ring which has three chiral centers with a single *RRR* stereoisomer (Figure 2). The chromanol groups have two fused rings, a phenol and a tetrahydropyran, sharing a 2 carbon bridge [17]. These rings are moderately polar, giving them an affinity for the cellular membrane surface while the phytyl tail is hydrophobic and normally associated with membrane lipids [18]. These structural features of *RRR*-α-tocopherol are efficiently acted on by the human hepatic α-TTP which is responsible for maintaining plasma α-tocopherol concentrations [19].

The synthetic α-tocopherol called all-racemic-α-tocopherol is not identical to *RRR*-α-tocopherol. It is an equimolar mixture of eight stereoisomers which possess three chiral centers at positions 2’, 4’, and 8’, giving rise to four diastereoisomeric pairs of enantiomers such as *RRR, RSR, RRS, RSS, SRR, SSR, SRS*, and *SSS*[20]. Moreover, α-TTP has a high affinity to *RRR*-α-tocopherol and has a 3-fold greater binding half-life when compared to synthetic α-tocopherol [21]. Thus, the bioactivity and the relative safety are different. Human proteins such as enzymes and receptors usually exhibit high stereospecificity [20]. Therefore, the natural *RRR*-α-tocopherol is more bioactive than the synthetic form.

*RRR*-α-tocopherol plays a major role as an antioxidant which prevents lipid peroxidation. In the photosynthetic cells, it may protect photosystem II during photoinhibition and repair chloroplast mechanisms [18]. This is due to the hydroxyl group on the C-6 position which is the active site that donates a hydrogen atom. The phenolic hydrogen atom is capable of scavenging lipid peroxy radicals and quenching singlet oxygen [22]. *RRR*-α-tocopherol is recycled in the photosynthetic cell by cytosolic ascorbate which oxidizes one-electron from the tocopheroxyl radical thus regenerating vitamin E [23]. This mechanism might protect the cell membranes.

*RRR*-α-tocopherol is biosynthesized in photosynthetic cells via two different pathways [24]. The phytyl domain precursor comes from an isoprenoid pathway, and the chromanol domain precursor comes from an alternative shikimate pathway homogentisic acid via complex enzymatic reactions [25]. *RRR*-α-tocopherol is found in the chloroplast envelope, thylakoids, and plastoglobuli of the plastid. The vitamin E biosynthetic pathway has been elucidated in Arabidopsis [26]. The genes associated with vitamin E biosynthesis in photosynthetic organisms have been well described in literature [3,27]. A significant metabolic engineering effort has been made to improve vitamin E content both in plants [28] and in cyanobacteria [29,30]. Moreover, tocopherol production in plant green callus [31], cell [32], and root cultures [15] have also been reported.

### *RRR*-α-tocopherol production in *S. bacillaris* strain siva2011

Photosynthetic algae are a potential alternative for production because they biosynthesize an abundance of *RRR*-α-tocopherol. For instance, freshwater *Euglena gracilis*[33], marine *Dunaliella tertiolecta* and *Tetraselmis suecica*[34], model system *Chlamydomonas reinhardtii*[35], and commercial algae *Spirulina platensis*[36] have been used to biosynthesize *RRR*-α-tocopherol. The new green alga, *S. bacillaris* strain siva2011 [16], produces significant amounts of *RRR*-α-tocopherol. This microalga has efficient photosynthetic mechanisms which facilitate the quick biosynthesis of vitamin E.

Tocopherol production can be enhanced by molecular elicitation which is non-transgenic. MeJa is a plant stress volatile signaling molecule which up-regulates several defense-related genes [37]. In plants, jasmonates are biosynthesized via the octadecanoid pathway; exogenous MeJa treatment is up-regulating secondary metabolic pathway genes especially those encoding for stress protection [38]. Therefore, it is used as one of the potential molecular elicitors in plant root culture to enhance several pharmaceutical molecule productions [15]. For instance, MeJa elicitation increases the activity of tyrosine aminotransferase in plant green cell culture which is one of the initial step enzymes involved in tocopherol biosynthesis [31,39]. To increase *RRR*-α-tocopherol content in *S. bacillaris* strain siva2011, MeJa elicitation was also used. The *S. bacillaris* strain siva2011 characteristics, culture conditions, and bioreactor experimental designs were previously reported for lipid production [16].

**Figure 3:**
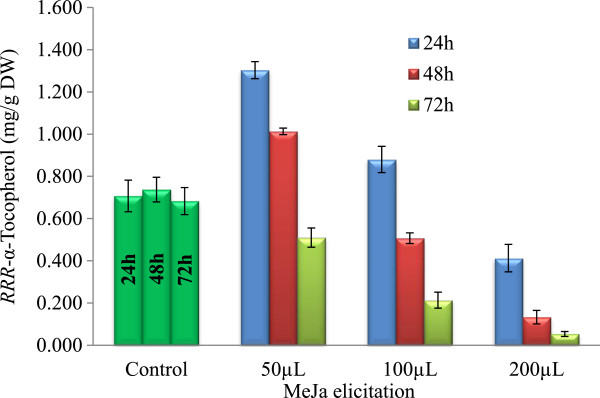
***RRR*-α-tocopherol content in *S. bacillaris *** **strain siva2011, unelicited and methyl jasmonate elicited culture on day 4, 5, and 6.**

Figure 3 illustrates the *RRR*-α-tocopherol content of *S. bacillaris* strain siva2011 biomass under elicitation with various concentrations of MeJa. The unelicited culture accumulated 0.7 mg/g DW of *RRR*-α-tocopherol which was detected starting during the early exponential growth phase. The lower concentration of MeJa, 50 μL/L, after 24 hrs elicitation, enhanced the production of *RRR*-α-tocopherol to the highest concentration, 1.3 mg/g DW. The higher MeJa concentrations or longer elicitation periods were inhibiting both to the biomass growth and the resultant *RRR*-α-tocopherol production. MeJa can diffuse to cells either by intercellular migration or while in the vapor phase [40]. In plants, MeJa can transport from leaves to roots [41]. The vapor signaling can be transported to distal plants via air, and the intercellular signaling can be transported via vascular process [42]. For instance, in an *in vitro* root culture the MeJa elicitation could trigger the defensive molecules accumulation via intercellular transport [13-15], whereas in *in vivo* plants during herbivore attack it can act as a volatile signal [42]. In the algal culture, MeJa elicitation could trigger the *RRR*-α-tocopherol production by either intercellular signaling or both. The optimum concentration can up-regulate the tocopherol biosynthetic pathway enzymes in *S. bacillaris* strain siva2011 which could increase the antioxidant. MeJa elicitation rapidly activates the defensive genes which also down regulates the photosynthetic system genes [38]. In addition, MeJa induces reactive oxygen species (ROS) which alter the mitochondrial and chloroplast dynamics [43]. Thus, the higher MeJa concentrations or longer elicitation periods can produce uncontrolled ROS which can precede chloroplast or photosynthetic dysfunctions which could be inhibiting the tocopherol biosynthesis and biomass accumulation in *S. bacillaris* strain siva2011. In green cells, chloroplasts are an essential organelle for energy capture and transduction; a decline in photosynthetic activity is closely related to the decrease in the biomass. The typical MeJa elicited cells’ symptoms were loss of chlorophyll, which causes the decline in the net photosynthetic rate, and degradation of ribulose bisphosphate carboxylase, etc. [44]. For instance, 100 μL MeJa at 9 hrs elicitation altered chloroplast morphology and function which is associated with cell death [43]. Even though *RRR*-α-tocopherol indirectly regulates the amounts of jasmonic acid [18], the decline in chloroplast efficiency could down regulate the *RRR*-α-tocopherol metabolism. This suggests that higher MeJa concentrations or longer elicitation could be cytotoxic beyond what was studied.

### Nonlinear regression

**Figure 4:**
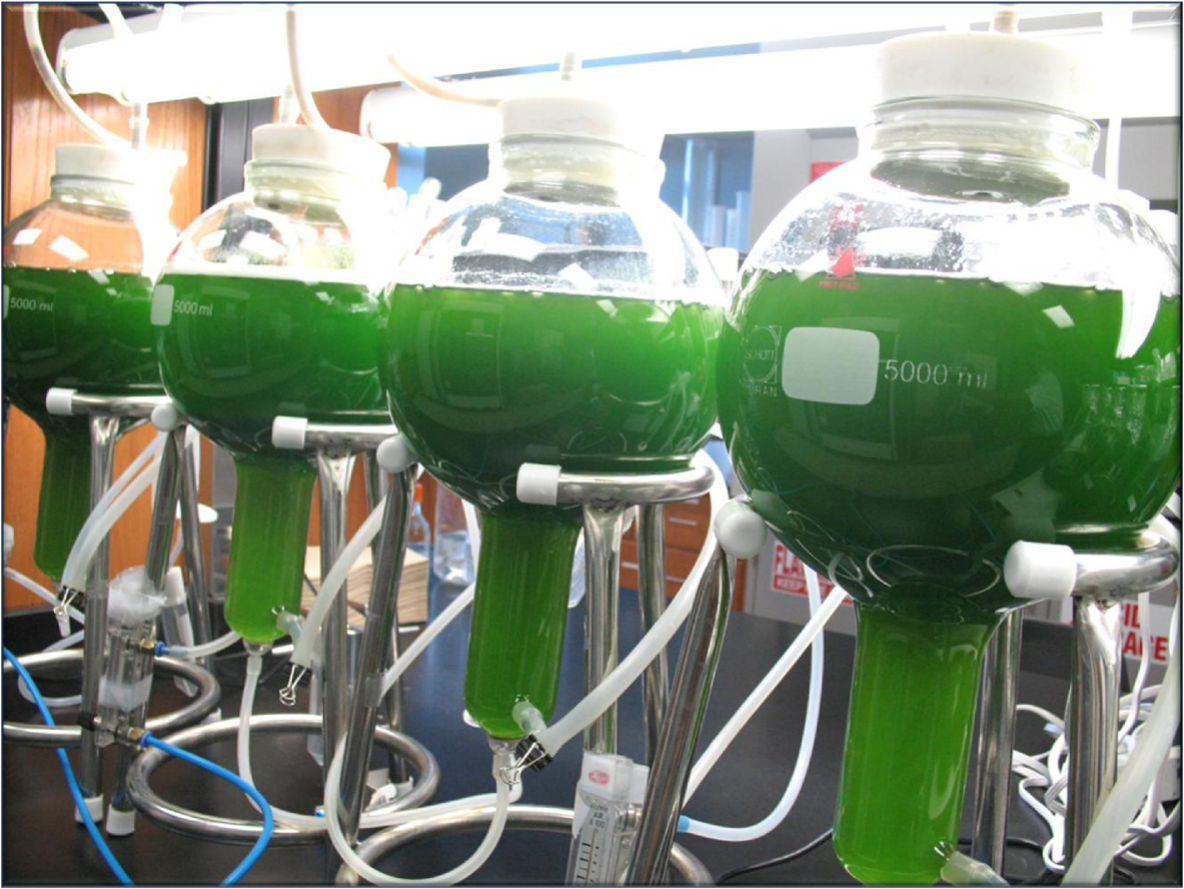
**Biomass production of *S. bacillaris *** **strain siva2011 in 5 L balloon type bioreactors (working volume 4 L).**

Compared to green adventitious roots, photosynthetic algae have a higher ability to biosynthesize *RRR*-α-tocopherol in bioreactor cultures. The balloon bioreactor is a liquid-phase reactor with enhanced geometry and efficient fluid flow dynamics which could help provide higher mass transfer efficiency [14]. When compared to other photobioreactors, the balloon bioreactor had a larger headspace which efficiently captured light and enhanced photosynthesis [16]. Thus, *S. bacillaris* strain siva2011 was scaled-up in a balloon bioreactor (Figure 4) to investigate enhanced biomass accumulation. Of the four concentrations of CO_2_ tested, 0.2% yielded the highest biomass of 3.45 g/L in 4 L and 3.79 g/L (DW) in 8 L on day 6 [16]. The *RRR*-α-tocopherol production was unchanged by the 0.2% CO_2_ (data not shown).

**Figure 5:**
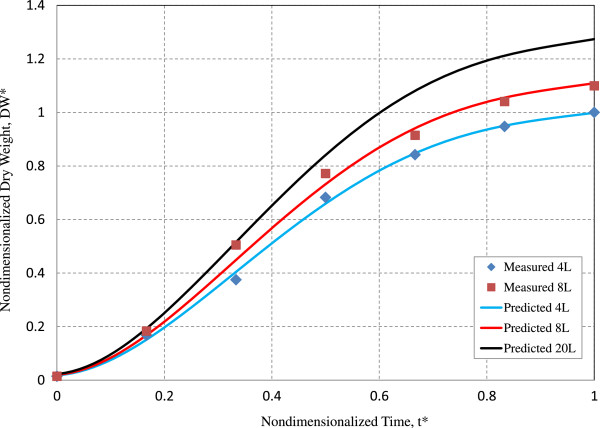
Nonlinear modeling for nondimensionalized dry weight from 4 L to 20 L.

To quantitatively predict larger-scale algal biomass production based on lab-scale test data, the following stepwise structured approach was proposed [45,46]: 1) to set up the simplest model to linearize with dual variables; 2) to model a nonlinear regression with dual variables; and 3) to demonstrate multiple variables based on a nonlinear regression. Although scale-up predication requires multiple variables, the 6 days algal culture does not significantly utilize all the media components. Therefore, selection of important dual variables can give insight on the efficiency of parameter selection for lab-scale validation and initial scale-up prediction. In addition, the multiple linear regression models might not incorporate the underlying nonlinear relationships [47]. So, in this study the second systematic approach was conducted to evaluate nonlinear modeling for scale-up prediction in a 20 L balloon reactor using dual variables. Nonlinear regression models are more important tools than linear models because they provide better parsimony, interpretability, and prediction [48]. Figure 5 illustrates nonlinear modeling of predicted *S. bacillaris* strain siva2011 biomass accumulation in a 20 L balloon bioreactor with 0.2% CO_2_. This model was generated using 4 L to 8 L data of *S. bacillaris* strain siva2011 and shows only small discrepancies between measured and predicted data. The nondimensional DW* can be converted into dimensional DW in g/L by multiplying by the maximum DW experimentally obtained from the baseline test which in this case is 4 L. The nonlinear modeling agrees with measured data both qualitatively and quantitatively, where the modeling has enhanced the R^2^ value up to 95% compared to the linear model value 87.4% [16]. This suggests that the nonlinear regression approach enhances accuracy of modeling which provides key scale-up informations of *S. bacillaris* strain siva2011 biomass for *RRR*-α-tocopherol production. In this study, the significance of this empirical approach provides insights into the application of a nonlinear regression model that increases the R^2^ value and enhances the quantitative predication of *S. bacillaris* strain siva2011 scaled-up in a 20 L bioreactor. This allows for the systematic understanding and design of a multi-variable nonlinear regression experiment for significant biomass production.

## Conclusions

Photosynthetic microalgae are rich in *RRR*-α-tocopherol and a potential source for this natural antioxidant which is an essential human micronutrient. A significant advantage of this natural source is the maintenance of the specific bioactive form needed for nutrition and the elimination of possible issues with potential unknown or unexpected toxicities from the synthetic conformations. *S. bacillaris* strain siva2011 has a unique vitamin E biosynthetic mechanism capable of sustaining high levels of production, including inducible enhanced production which could provide a possible production platform of *RRR*-α-tocopherol for pharmaceutical industries. A nonlinear mathematical model was developed to model scale-up to production in 20 L reactors using an approach with a higher accuracy based on the dual variables tested in 4 L and 8 L reactors. The R^2^ value from this study demonstrates that this nonlinear approach significantly improves estimation of *S. bacillaris* strain siva2011 biomass production in the bioreactor than does the linear model. Additional studies with progressively larger reactors (and models) will be needed to bridge the gap between laboratory and industrial scale. Nevertheless, this data provides enhanced bioprocess engineering information in the progression towards large-scale pharmaceutical *RRR*-α-tocopherol production from *S. bacillaris* strain siva2011 biomass.

## Methods

### Bioreactor culture, elicitation and analytics

*S. bacillaris* strain siva2011 cells were cultured in a balloon type bioreactor (4 L and 8 L). The bioreactor experimental design and the biomass harvesting were performed as described by Sivakumar et al. [16]. For elicitation, three concentrations of filter sterilized MeJa 50, 100, and 200 μL/L were added to the *S. bacillaris* strain siva2011 culture on the 3^rd^ day. MeJa dissolved in ethanol and MeJa not dissolved in ethanol were tested, and both had a similar effect. Elicitations were carried out for 24, 48, or 72 hrs. Algal cells were harvested and freeze-dried according to the Sivakumar et al. [16] method. One gram of MeJa elicited and unelicited freeze-dried algal cells were used for analysis of *RRR*-α-tocopherol. *RRR*-α-tocopherol was processed according to the Sivakumar et al. [4] method. The reversed phase-high performance liquid chromatography chromatogram was acquired using a Dionex 3000 (LPG3400A) system (Thermo Scientific, Sunnyvale, CA) equipped with a thermostatted UltiMate 3000 autosampler and a Dionex RF 2000 fluorescence detector. The system was monitored by the software’s chromeleon (6.80) for instrument control and data acquisition, data reprocessing, and solute quantification, respectively. An Agilent Zorbax Eclipse XDB-C8 (250 mm × 4.6 mm, mean particle size 5 μm) column or C18 (250 mm × 4.6 mm, mean particle size 5 μm) was used to separate *RRR*-α-tocopherol. The mobile phase consists of a linear gradient of 90% methanol in water. The flow rate was 1 ml/min. The total acquisition time was 35 min. The wavelength was set at 290 nm for excitation and 330 nm for emission. The authenticated *RRR*-α-tocopherol fluorescence spectra and retention time was used for HPLC confirmation of *RRR*-α-tocopherol in the samples. The *RRR*-α-tocopherol liquid chromatography mass spectrometry spectrum confirmation was acquired using a Shimadzu 8050 mass spectrometer.

### Nonlinear regression

For a stepwise approach, the first phase of the nonlinear regression method begins with the simplification from five variables to two variables, time (t) and reactor volume (V), as shown in equation 1. The regression will be extended to multivariables when the two variables method is validated.

(1) $$\mathrm{DW}=f\;\left(y_{\mathrm{CO}2},\mathrm{EC},\mathrm{OrP},\mathrm{pH},t,V\right)\approx f\;\left(t,V\right)$$

The associated variables DW, t, and V in equation 1 are nondimensionalized into DW*, t*, and V* as shown in equation (2) to (4).

(2) $$DW^{*}=\frac{\mathrm{DW}}{DW_{\max}}$$

(3) $$t^{*}=\frac{t}{t_{\max}}$$

(4) $$V^{*}=\frac{V\;\left[L\right]}{4L}$$

Where DW_max_ is the maximum DW [g/L] produced from 4 L test under 0.02% CO_2_ fraction, and t_max_ is the maximum time to reach the maximum DW. In this study, DW_max_ and t_max_ were 3.45 g/L and 6 days, respectively. The volume was standardized by the baseline: 4 L in this modeling.

A nonlinear model was assumed by combining 4^th^ order polynomials and the power form of the equation as shown in equation (5) in consideration of measured data of 4 L and 8 L.

(5) $$DW^{*}=\left(a{t^{*}}^{4}+b{t^{*}}^{3}+c{t^{*}}^{2}+d{t^{*}}^{2}+\mathrm{et}+f\right){V^{*}}^{g}$$

All associated constants a, b, c, and d in equation (5) were determined from a nonlinear regression method as shown in equation (6).

(6) $$\begin{array}{ll} DW^{*}= & \left(2.686{t^{*}}^{4}-6.942{t^{*}}^{3}+5.109{t^{*}}^{2}\right)\\ & \left(+0.127t+0.0184\right){V^{*}}^{0.151} \end{array}$$

Equation (6) allows the predicting of DW in reactor volume 20 L at 0.02% CO_2_ fraction from the correlation obtained from 4 L and 8 L measured data.

### Statistical analysis

Bioreactor culture, elicitations, and analytical experiments were repeated at least three times, each with three replications. Results were expressed as the mean with standard errors. Stepwise dual nonlinear regressions were used to investigate the relationship between 4 L and 8 L in order to predict 20 L bioreactor scale-up.

## Abbreviations

C: Carbon; CO_2_: Carbon dioxide; DW: Dry weight; G: Gram; hrs: Hours; α: Trimethyl; β, γ: Dimethyl; δ: Monomethyl; α-TTP: α-Tocopherol transfer protein; MeJa: Methyl jasmonate; Mg: Milligram; μL: Microliter; L: Liter; %: Percentage; ROS: Reactive oxygen species.

## Competing interests

The authors declare that they have no competing interests.

## Authors’ contributions

GS, JL, and KJ made substantial contributions to the experimental design, analysis, and/or interpretation of data. Specifically, GS led and designed the experiments and performed the bioprocessing and HPLC studies. JL performed the mass spectrometry experiments for compound confirmation. KJ performed the mathematical modeling to validate the variables. GS wrote the manuscript which was reviewed and approved by all authors. All authors read and approved the final manuscript.
